# Necroptosis Related Genes Predict Prognosis and Therapeutic Potential in Gastric Cancer

**DOI:** 10.3390/biom13010101

**Published:** 2023-01-04

**Authors:** Nandie Wu, Fangcen Liu, Ying Huang, Xinyu Su, Yaping Zhang, Lixia Yu, Baorui Liu

**Affiliations:** 1 The Comprehensive Cancer Centre of Nanjing Drum Tower Hospital, Clinical College of Nanjing Medical University, Nanjing 210009, China; 2 Department of Pathology, Drum Tower Hospital, Medical School of Nanjing University, Nanjing 210009, China; 3 The Comprehensive Cancer Centre of Nanjing Drum Tower Hospital, Clinical College of Traditional Chinese and Western Medicine, Nanjing University of Chinese Medicine, Nanjing 210009, China; 4 Department of Oncology, The Second People’s Hospital of Huai’an, Huai’an 223022, China; 5 The Comprehensive Cancer Centre of Nanjing Drum Tower Hospital, The Affiliated Hospital of Nanjing University Medical School, Nanjing 210009, China; 6 The Comprehensive Cancer Centre of Nanjing Drum Tower Hospital, Clinical College of Xuzhou Medical University, Nanjing 210009, China

**Keywords:** gastric cancer, necroptosis, immune cell infiltration, immune microenvironment, glycolysis, drug response

## Abstract

The clinical significance of necroptosis in gastric cancer (GC) has yet to be fully elucidated. The purpose of our study was to identify a necroptosis-relevant gene and to establish a prediction model to estimate the prognosis and therapeutic potential in GC. Here, we explored the expression profile of 76 necroptosis-related genes in TCGA-STAD patients. A six-gene risk score prediction model was established via regression analysis of the least absolute shrinkage and selection operator (LASSO) and validated in a separate cohort. Patients were separated into low- or high-risk groups according to the median risk score. We then compared and analyzed the biological process characteristics of two risk groups. Additionally, cell-to-cell communications and metabolic activity were analyzed in a single-cell solution. The in vitro experiments were conducted to explore the biological functions and drug sensitivity of necroptosis-related genes in gastric cancer. Our results identified that compared with the low-risk group, the high-risk group was associated with a higher clinical stage or grade and a worse prognosis. In addition, the low-risk group had higher levels of immunity and immune cell infiltration. Necroptosis was triggered by the TNF pathway in myeloid cells and the glycolysis pathway was altered. Necroptosis-related genes modulated the cell function, including proliferation and migration in vitro. Furthermore, the potential drugs’ sensitivity was higher in the low-risk subgroup. These findings could facilitate a better understanding and improve the treatment potential and prognosis of GC patients.

## 1. Introduction

Gastric cancer (GC) is a complex and heterogeneous disease and is the third most common cause of cancer death worldwide [1]. Although chemotherapy and targeted therapy based on novel molecular characteristics have proven effective in GC, the 5-year overall survival (OS) rate of advanced GC is still less than 40% [2]. Identification of carcinogenesis pathways and relevant driver genes could contribute to the treatment of GC, and these pathways may ultimately become therapeutic targets.

Cell death has both physiological and pathological functions. Various forms of cell death have been identified thus far, such as apoptosis, necroptosis, and pyroptosis [3]. Nevertheless, therapeutic agents targeting cell death-related genes such as BCL-2 family proteins have poor effects in antitumor therapy [4]. Necroptosis is a newly discovered type of programmed cell death that is characterized by pro-inflammatory markers and is caspase independent [3]. The significant role of necroptosis in tumors has been preliminarily explained. Necroptosis has been suggested for the regulation of cancer biology, including oncogenesis and cancer invasion [5]. The induction of cell death mechanisms including necroptosis has been recognized as a promising field of cancer therapy [6]. Necroptosis is strongly associated with cancer prognosis. Necroptosis-related lncRNAs could predict prognosis and help differentiate between cold and hot tumors in GC [7]. The expression of the necroptosis-related gene RIPK3 was suggested to be an independent prognostic factor of overall survival and disease-free survival in colorectal cancer patients according to Cox proportional risk model analysis [8]. Downregulation of the expression of necroptosis key molecules RIPK3 was found to independently reduce OS in cancer [8,9].

Immunotherapy has revolutionized the therapeutic methods of oncology including GC [1,10]. In CheckMate-649, a combination of nivolumab and chemotherapy demonstrated superior OS compared to chemotherapy alone, regardless of PD-L1 expression [11]. Most recently, the Phase III trial KEYNOTE-859 [12] achieved significantly longer OS when patients were treated with pembrolizumab and chemotherapy compared to chemotherapy alone. However, a portion of GC patients showed natural resistance to checkpoint inhibitors. It was reported that inflammatory cell death could activate anticancer immunity [13]. In 2016, Aes et al. reported that necroptosis of tumor cells could trigger antigen presentation and thus activate cytotoxic CD8+ T lymphocytes [14]. Necroptosis could recruit inflammatory cells of the immune system [15]. Meanwhile, crosstalk was identified between necroptosis and antitumor immunity [16]. Furthermore, necroptosis proved to play a vital role in the immune microenvironment [13]. Altered energy metabolism was reported as the next-generation hallmark of cancer [17]. Elevated glycolysis metabolism in activated immune cells could reprogram immune functions from multiple aspects [18]. However, the crosstalk between necroptosis and metabolism pathways has yet to be fully elucidated.

Hence, it is pertinent to identify biomarkers associated with necroptosis which can predict prognosis, activate anticancer immunity, and improve the outcomes of GC patients. The clinical value of this necroptosis-related gene signature was also explored in the immune microenvironment, metabolic status, and drug sensitivity of GC. Our study aims to provide a baseline for the treatment of GC.

## 2. Materials and Methods

### 2.1. Data Acquisition and Processing

Gene expression and clinical data were obtained from The Cancer Genome Atlas (TCGA-STAD) database for the identification of differently expressed genes and training cohort of the risk signature. Data of GSE84437 from the Gene Expression Omnibus database validated the established risk signature. Differential expressed gene (DEG) analyses of necroptosis-related genes (NRGs) between tumor samples and their non-tumor counterparts were conducted with the “limma” package. NRG genes with an adjusted p-value less than 0.05 and |log fold change (FC)| greater than 1.5 were determined as differentially expressed NRGs. A comparison of the clinical–pathological characteristics between the TCGA-STAD cohort and GSE84437 cohort is provided in Table S1.

### 2.2. Establishment and Verification of Necroptosis-Related Risk Signature

We began by retrieving 76 NRGs from MSigDB and previous studies [19,20,21]. A two-stage procedure was employed to build the prognostic risk score model. First, NRGs that were differentially expressed between normal and tumor samples in TCGA-STAD cohort were retrieved. Then, the least absolute shrinkage and selection operator (LASSO) Cox regression analysis was used to select the candidate genes and develop a necroptosis-related prognostic signature using the “glmnet” package. The corresponding coefficient of each candidate gene was obtained to calculate the risk score of each TCGA-STAD patient using the following formula:

risk score $=\overset{n}{\underset{i=1}{\sum }}exp^{Gene\;i}coef^{Gene\;i}\;$(exp: candidate gene expression, coef: coefficients)

Patients were assigned to a low- or high-risk group according to the median score. Survival curves of Kaplan–Meier analysis were generated using the “survminer” and “survival” packages. The “timeROC” packages were used to create time-dependent ROC curves. Furthermore, the independent cohort (GSE84437, N = 433) was included for external validation due to its similar calculation and stratification.

### 2.3. Functional Enrichment and Pathway Enrichment Analysis for Subgroups

GO analysis of differentially expressed genes between two NRG risk subgroups was performed by the “clusterprofiler” package in R4.0.5. Gene Set Variation Analysis (GSVA) was also performed to determine the relative enrichment of gene sets in KEGG database between two subgroups.

### 2.4. Construction of Nomogram

The prognostic values in the multivariate regression analysis with statistical significance were selected for the construction of nomogram with “regplot”, “survival” and “survminer” packages. The decision curve analysis (DCA) was performed to demonstrate the prognostic prediction performance of nomogram. Calibration curves were created using the package “rms” to assess the accuracy for 1- and 3-year OS prediction of the nomogram.

### 2.5. Mutation Patterns of Two Subgroups

Copy number variations (CNVs) and somatic mutation data of TCGA-STAD were obtained from GDC Data Portal (Genomic Data Commons) (https://portal.gdc.cancer.gov/, accessed on 25 March 2022). The R package “maftools”, “sigminer” and “MOVICS” were utilized to explore the genetic landscape and compare differences in somatic mutation between two NRG risk subgroups. The online GISTIC 2.0 module of GenePattern was utilized to identify amplified or deleted broad and focal segments of the genome.

### 2.6. Gene Set Enrichment Analysis

Gene Set Enrichment Analysis (GSEA) was conducted to identify significantly enriched gene sets between two risk groups. The reference set was c5.go.v7.5.1.symbols.gmt.

### 2.7. Immune Infiltration Analysis

The immune score and stromal score based on the gene expression pattern between two groups were calculated using the R package “estimate”. TIMER2.0 database was applied to evaluate the abundance of the six immune infiltration cells in TCGA-STAD patients. Single-sample gene set enrichment analysis (ssGSEA) was performed to estimate and compare estimate the infiltration abundance of immune cells and immune-related pathways in the high- and low-risk groups. The packages “limma” and “ggpubr” were used to visualize the results. Furthermore, the relative infiltration of 28 immune cell types together with macrophage subpopulations based on ssGSEA algorithm and tumor immune infiltration using the CIBERSORT algorithm were presented via heatmap.

### 2.8. Single Cell Data Processing and Cell–Cell Interaction Analysis

The single-cell sequencing data of GSE163558 were downloaded. Data processing was performed using “Seurat”. Cells with <10% mitochondrial genes and those containing genes ranging from 200 to 7000 were retained. The “harmony” method was used for sample integration. Dimension reduction of data was performed using the tSNE method. All the clusters were annotated with the R package “scHCL”. Cell–cell interaction analysis was conducted with the software “cellphonedb” and in miniconda (https://anaconda.org/, accessed on 11 March 2022) and the R package “CellChat”.

### 2.9. Analysis of Single-Cell Glycolysis Activity

T cells were reserved, and the main clusters of T/NK cells were defined with commonly used cell markers (CD4+T cells: CD4; CD8+T cells: CD8A; Tregs: FOXP3; NK cells: KLRC1). Single-cell metabolic activity was quantified using the R package “scMetabolism”. The average metabolic gene expression and median metabolic pathway score of four cell types were evaluated. Single-cell rank-based gene set enrichment analysis of HALLMARK-GLYCOLYSIS pathway was performed using the R package “irGSEA”.

### 2.10. Cell Culture and RNAi

The human gastric cancer cell lines SNU601, SNU668, MKN45, and NUGC-4 were obtained from the National Cancer Center Research Institute (Tokyo, Japan) in 2011. KATO-III, NCI-N87, AGS, and HGC27 cell lines were purchased from the Chinese Collection of Research Bioresources (Shanghai, China). The cells were cultured in RPMI-1640 medium (Corning, NY, USA) supplemented with 10% fetal bovine serum (Gibco, Carlsbad, USA) at 37 °C in a humidified atmosphere of 5% CO2. For siRNA transfection, gastric cancer cell lines were seeded into six-well plates and transfected with either non-targeting control siRNA (si-NC) or a short interfering RNA (Genepharma, Shanghai, China) targeting candidate NRGs (si-KLF9, si-TNFRSF1B, si-BCL2L11, si-PLK1, si-HSPA4, and si-TSC1) 24h after seeding using Lipo3000 transfection reagent (Invitrogen, Carlsbad, USA) according to the manufacturer’s instructions. The cells were then incubated for a further 48 h prior to collection. The sequences of siRNAs used are described in Table S2.

### 2.11. Cell Proliferation Assay

Transfected cells were seeded in 96-well plates (5 × 103 cells/well) and cultured in 200 μL culture medium. Cell Counting Kit-8 (CCK-8) assay was carried out in line with the manufacturer’s protocol (NCM Biotech, Suzhou, China) at 24, 48, 72, and 96 h time points after transfection. After 30 min of incubation, the absorbance of each well was measured using a microplate reader at a wavelength of 450 nm. Each experiment was performed in triplicate.

### 2.12. Monoclonal Formation

After transfection, gastric cancer cells were trypsinized, serially diluted, and plated into six-well plates containing 400 cells per well. After 14 days of incubation, cells were fixed and stained with 0.25% methylene blue (Beyotime, Beijing, China) for counting.

### 2.13. Wound Healing Assays

Gastric cancer cell lines were seeded into six-well plates after transfection. Cells were cultured to >90% confluency and then the plate was scratched with a sterile 200 μL pipette tip. Images were taken 0, 12, 24, and 48 h after scarring.

### 2.14. RNA Extraction and Quantitative Real-Time PCR

Total RNA was extracted from the cultured cells and human tissues using TRIzol reagent (Invitrogen), and first-strand cDNA was synthesized using the First-Strand cDNA Synthesis Kit (Thermo Fisher Scientific, Inc., Waltham, USA) according to the manufacturer’s protocols. For mRNA expression detection, SYBR Green qPCR Master Mix (Thermo Fisher Scientific, Inc., Waltham, USA) was used. The mRNA expressions were detected on ABI QuantStudio 7 Flex real-time PCR system (Applied Biosystems, Waltham, USA). The relative levels of mRNAs in cells and tissues were normalized to the housekeeping gene GAPDH and calculated with Equation 2^−ΔΔCT^. The primers used are described in Table S3.

### 2.15. The In Silico Drug Sensitivity Analysis and In Vitro Drug Dose Response Investigations

The predicted relationship between mRNA expression of risk genes and GDSC drug sensitivity of TCGA-STAD patients was derived from the GSCA online database. Furthermore, using R package “MOVICS”, 161 prodrug prodrugs with therapeutic potentialities were investigated to predict IC50 values based on the gene expression pattern between the two groups. To validate the results of in silico drug-sensitivity analysis, eight gastric cancer cell lines were subsequently seeded in 96-well plates. Cells were treated with increasing dosages of selected drugs (drug list in Table S4) and then incubated for 48 h. Cell viability was evaluated with CCK-8 assays, as previously described.

### 2.16. Statistical Analyses

One-way ANOVA was performed to compare the differences in NRG expression in the TCGA cohort. The Mann–Whitney–Wilcoxon test was used for between-group comparisons of TMB, immune infiltration, and predicted IC50 values. All data analysis and visualization were performed with R 4.0.5. The IC50 of in vitro drug dose–response experiments was analyzed with Prism v7.0. *p* values < 0.05 were considered statistically significant. All *p*-values were two-sided.

## 3. Results

### 3.1. Establishment of Necroptosis-Related Prognostic Signature

To establish the necroptosis-related prognostic signature of gastric cancer, we first explored the RNA-sequencing data of the TCGA-STAD cohort containing 36 normal and 408 tumorous stomach samples. Gene differential analysis of 76 NRGs was evaluated. As a result, 57 NRGs were differentially expressed between normal and tumor samples (Figure S1A). These genes were further utilized with LASSO Cox regression analysis to construct a prognostic classifier, from which we chose six optimum genes to construct a prognostic signature (Figure S1B,C), four risk lncRNAs (KLF9, BCL2L11, PLK1, and HSPA4) with HR > 1 and two protective lncRNAs (TNFRSF1B and TSC1). HR < 1 revealed the coefficient and partial likelihood deviance of the prognostic signature.

**Figure 1:**
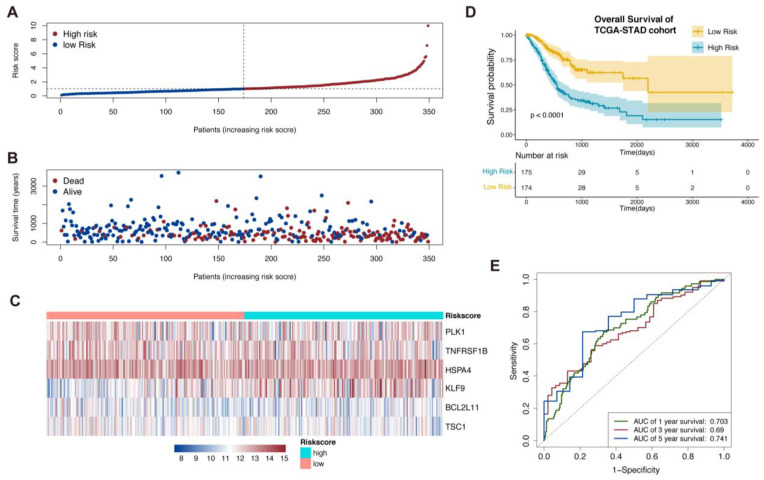
Establishment of necroptosis-related prognostic signature. (**A**) TCGA-STAD patients sorted by risk score. (**B**) The correlation between the patient’s prognosis and the risk score. (**C**) A heatmap of expression levels of the 6 genes in prognostic signature. (**D**) Kaplan–Meier survival analysis of TCGA-STAD patients between low-risk and high-risk groups. (**E**) Time-dependent ROC analysis of necroptosis-related prognostic signature.

The risk score was calculated as follows: Risk score = (0.3522) * KLF9 + (−0.3687) * TNFRSF1B + (0.43379) * BCL2L11 + (0.4306) * PLK1 + (0.5425) * HSPA4 + (−0.4971) * TSC1. When using the median value of risk score as the cut-off value, all patients could be divided into a high-risk and a low-risk group (Figure 1A). The low-risk patients were more likely to show a better prognosis from the results of the scatterplots (Figure 1B). The expression levels of these six genes showed distinct characteristics between two risk groups (Figure 1C). The Kaplan–Meier survival curve showed that patients with low-risk score had a better overall survival (OS) rate compared to those with a high-risk score (*p* < 0.001, Figure 1D). The sensitivity and specificity of the prognostic signature were further evaluated from the ROC curves by calculating the areas under the curve (AUC). A favorable result of predicting the OS status was determined (1-year AUC = 0.703, 3- year AUC = 0.69, and 5-year AUC = 0.741) in Figure 1E.

The differential expressed genes and clinical–pathological features between two risk subgroups were detailed in Figure S2A. The patients with advanced T stage (*p* = 0.009), N stage (*p* < 0.001), and tumor stage (*p* = 0.010) showed significantly higher risk scores (Figure S2B). Biological functions in signature-related downstream pathways were explored with GSVA, GO, and KEGG pathways analysis. The results indicated that DEGs could be altered in several tumorigenesis-related molecular functions pathways and metabolism pathways. Pathways such as TGF-beta, focal adhesion, hedgehog signaling, ECM–receptor interaction, glycosaminoglycan biosynthesis, and pentose phosphate pathway were altered in the two risk groups (Figure S2C). Similarly, GO and KEGG analysis also showed that metabolic-related signaling, such as ATP-dependent transmembrane transport complex and glutamate receptor signaling, were enriched (Figure S2D,E).

### 3.2. Validation of the Necroptosis-Related Prognostic Signature

**Figure 2:**
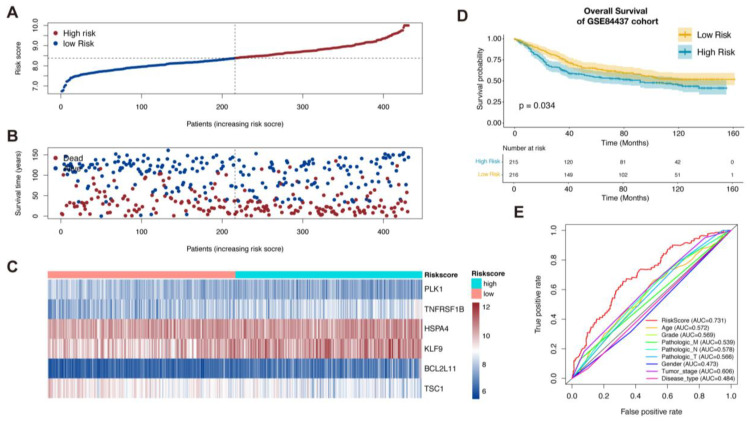
Validation of the necroptosis-related prognostic signature in GSE84437 and establishment of nomogram. (**A**–**C**) Patients in GSE84437 were sorted by risk score according to the necroptosis-related prognostic signature. (**D**) Kaplan–Meier survival analysis of GSE84437 patients to validate the necroptosis-related prognostic signature. (**E**) Comparison ROC analysis between necroptosis-related prognostic signature and the clinical factors.

To validate the signature, we used the GSE84437 dataset as the validation cohort. Based on the median risk score, the patients’ validation cohort could also be sorted into the high-risk group and low-risk group. Similar to the TCGA cohort, low-risk patients had a higher probability of a better prognosis than those with high-risk scores (Figure 2A–D). ROC curves indicated that risk scores presented the best potential to predict the OS status compared to other clinical pathology factors (Figure 2E).

### 3.3. Analysis of Independent Prognostic Factors

**Figure 3:**
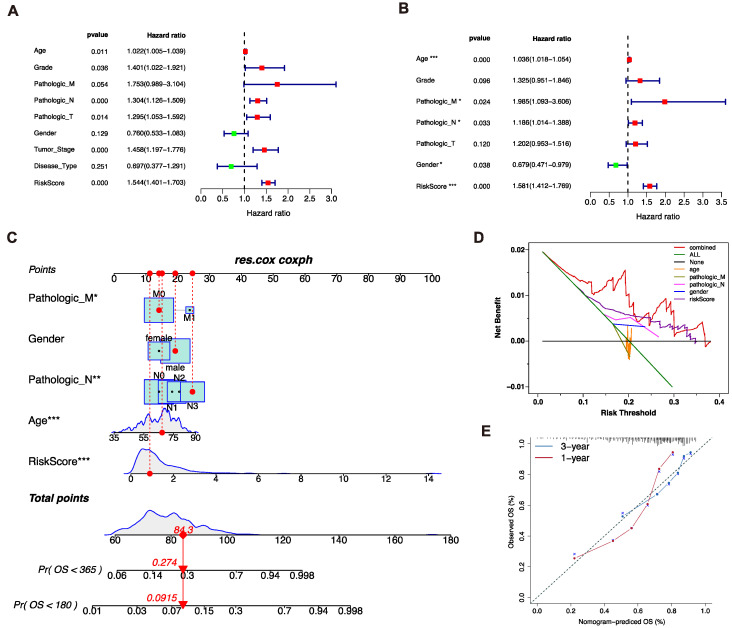
Establishment of nomogram. (**A**,**B**). Univariate and multivariate Cox regression analysis of clinical factors and risk score for overall survival. (**C**). Independent prognostic factors in multivariate Cox regression analysis for construction of Nomogram. (**D**). Decision curve analysis for comparison of multiple models to predict the overall survival of patients. (**E**). Calibration curves of nomogram for 1 and 3 years (*, ** and *** = significant at 0.05, 0.01 and 0.001 levels, respectively)..

In order to elucidate whether the risk score was the independent prognostic factor, univariate and multivariate Cox regression analysis was applied in the TCGA cohort. The results of the univariate Cox regression analysis showed that the risk score (*p* < 0.001, HR = 1.544, 95%CI = 1.401−1.703) and other clinical parameters, including age (*p* = 0.011, HR = 1.022, 95%CI = 1.005−1.039), differentiated degree grade (*p* = 0.036, HR = 1.401, 95%CI = 1.022 -1.921), distant metastasis (*p* = 0.054, HR = 1.753, 95%CI = 0.989−3.104), N stage (*p* < 0.001, HR = 1.304, 95%CI = 1.126−1.509), T stage (*p* = 0.014, HR = 1.295, 95%CI = 1.053−1.592), and clinical stage (*p* < 0.001, HR = 1.458, 95%CI =1.197−1.776), were significantly associated with OS (Figure 3A). Furthermore, multivariate Cox regression analysis was conducted. The results indicated that the risk score (*p* < 0.001, HR = 1.581, 95%CI = 1.412−1.779), age (*p* < 0.001, HR = 1.036, 95%CI = 1.018−1.054), distant metastasis (*p* = 0.024, HR = 1.985, 95%CI = 1.093−3.606), N stage (*p* = 0.033, HR = 1.186, 95%CI = 1.014−1.388), and gender *p* = 0.038, HR =0.679, 95%CI =0.471−0.979) were the independent prognostic factors of the OS (Figure 3B). Thus, the risk score was validated as an independent prognostic factor of the OS.

### 3.4. Construction of the Nomogram Prediction Model

All independent prognostic factors were applied to construct the nomogram prediction model, which could predict the survival probability of GC patients (Figure 3C). DCA analysis indicated the nomogram prediction model presented the best potential ability to predict the survival probability compared to other factors (Figure 3D). The nomogram prediction model showed good reliability in the observations of 1−3 years survival rate (Figure 3E). In conclusion, the nomogram consisted of the clinical features and the risk score was stable and accurate.

### 3.5. Comparison of Integrated Genomic Signatures in the Two Rick Groups

**Figure 4:**
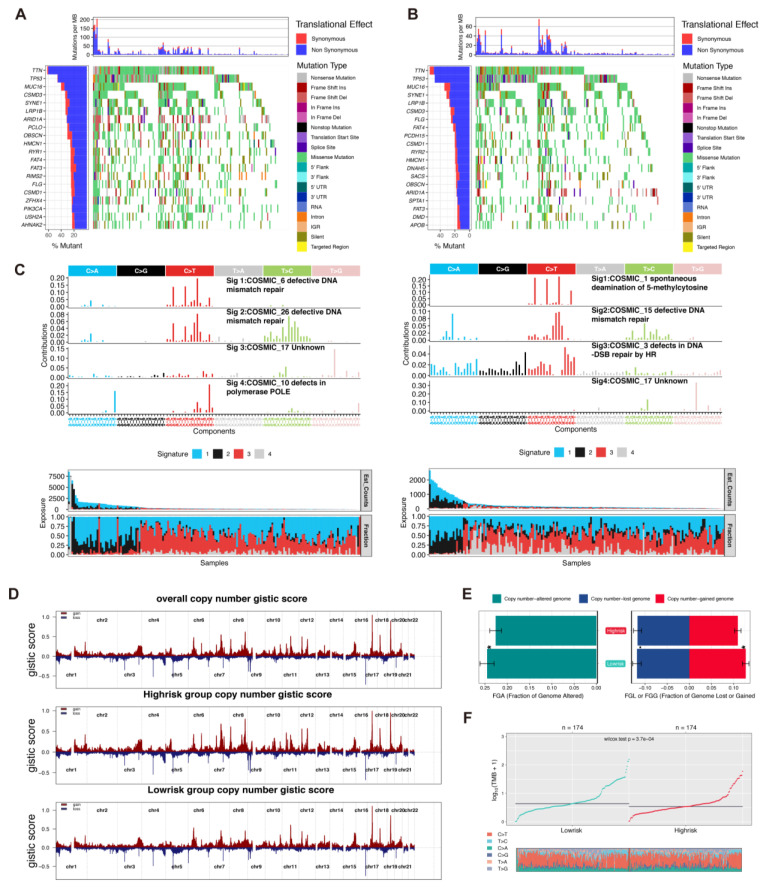
Genomic mutation patterns of two risk groups. (**A**,**B**). Somatic mutations of patients in low-risk and high-risk groups were displayed via waterfall plot. (**C**). The COSMIC SBS mutation signatures of two risk groups. (**D**). The genomic copy number variations of TCGA-STAD patients, analyzed by Gistic 2.0. (**E**,**F**). Comparison of TMB and fraction genome alteration between two subgroups.

The genomic disparity between the two risk subgroups was explored. The top 20 most frequently mutated genes are displayed in waterfall plots (Figure 4A refers to the low-risk group and Figure 4B refers to the high-risk group). Next, the analysis of single-base substitution (SBS) signatures from the COSMIC database showed that the single-nucleotide variants pattern of the low-risk group harbored signatures 6, 26, 17, and 10, while the high-risk group signatures exhibited signatures 1, 15, 3, and 17. Interestingly, signature 10 reflected defects in polymerase epsilon (POLE), which contributes to genomic stability during normal DNA replication (Figure 4C) [22]. Other than the mutation pattern, we also decoded the differences in the CNVs between the two subgroups. The GISTIC2.0 software was used to verify the CNVs on chromosomes. The recurrent CNVs in the low-risk group included the unique amplification of 3q26.32 (PIK3CA), as well as the deletions of 2q34 (ERBB4), 8p21.2 (FGFR1),12q23.1 (ERBB3, KRAS), and 1p33(NRAS) (Figure 4D). Meanwhile, the high-risk group included the unique deletion of 1p36.23 (mTOR). The CNVs in the low-risk group were mainly associated with the DNA damage repair pathway, such as the amplification of 13q14.11(BRCA2 and RFC3) and 1q21.3 (FASLG) (Figure 4D). Compared with the high-risk group, the low-risk group had a higher altered copy number in the genome and gained a higher copy number in the genome (Figure 4E). Furthermore, the tumor mutation burden (TMB) rate in the low-risk group was higher than that in the high-risk group (Figure 4F).

### 3.6. Immune Profile of the Risk Score and Necroptosis-Related 6 Genes

**Figure 5:**
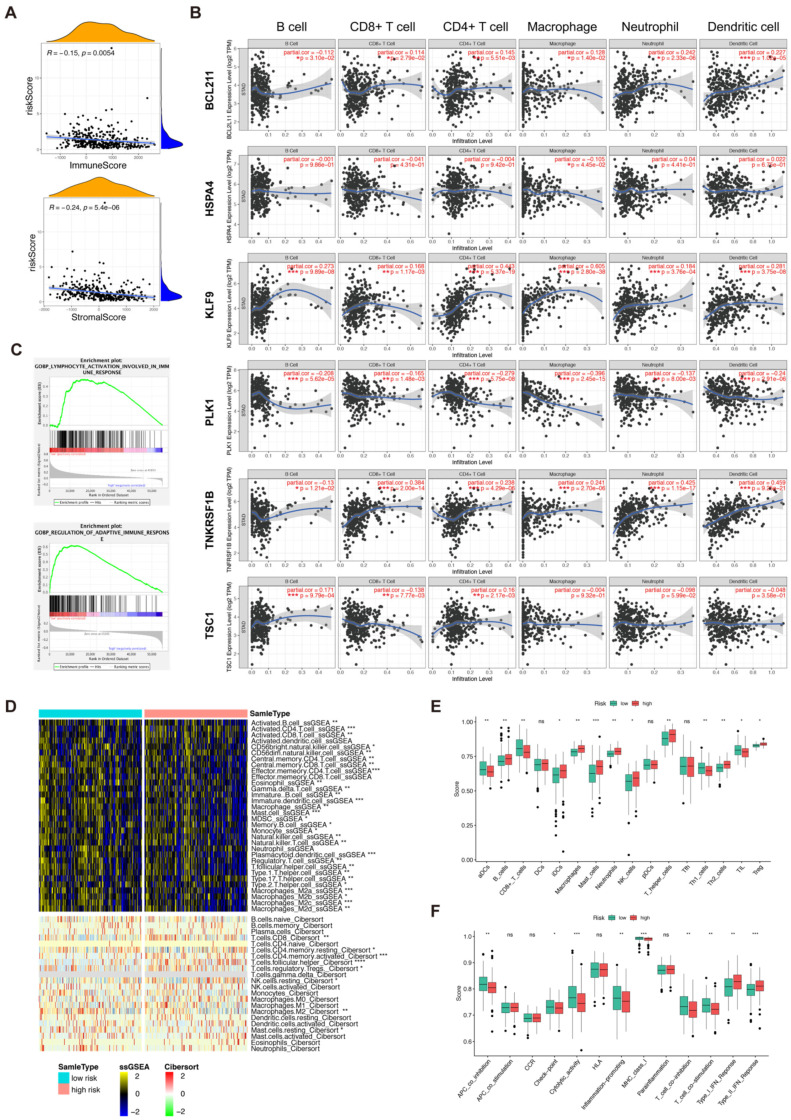
The immune landscape of gastric cancer patients. (**A**). Pearson correlation analysis of immune score and risk score. (**B**). The correlation analysis between 6 prognostic necroptosis-related genes expression and infiltration of six types of immune cells by TIMER 2.0 database. (**C**). GSEA analysis of immune-related gene sets. (**D**). GSVA analysis of immune infiltration based on CIBERSORT and ssGSEA algorithms. (**E**,**F**). The comparison of 16 immune cells and 13 immune-related pathways ssGSEA analysis between two subgroups (ns = non-significant; *, ** and *** = significant at 0.05, 0.01 and 0.001 levels, respectively).

The association between immune-related scores and risk scores was investigated using the ESTIMATE algorithm. As a result, the risk score was negatively related to the stromal score and immune score (*p* < 0.05, Figure 5A). These results highlight the heterogeneous intratumor immune microenvironment features of two risk subgroups. Since necroptosis possesses antitumor immunity, the correlation between NRGs and immune infiltration score was calculated by TIMER 2.0 (Figure 5B). HSPA4 was negatively correlated with macrophages (*p* = 0.045). TSC1 was negatively correlated with CD8+T cells (*p* = 0.008) but correlated positively with CD4+T cells (*p* = 0.002) and B cells (*p* < 0.0001). There was a significant positive correlation between KLF9 and B cells, CD8+T cells, CD4+T cells, macrophages, neutrophils, and DC (*p* < 0.05), while PLK1 had a negative correlation with such immune cells. In addition, BCL2L11 and TNFRSF1B expression level was positively correlated with CD8+T cells, CD4+T cells, macrophages neutrophils, and DC cells (*p* < 0.05), and negatively correlated with B cells (*p* < 0.05). Furthermore, GSEA analysis showed that the immune-related pathway including GOBP_REGULATION_OF_ADAPTIVE_IMMUNE_RESPONSE and GOBP_LYMPHOCYTE_ ACTIVATION_INVOLVED_ IMMUNE_RESPONSE was enriched in the low-risk group (Figure 5C).

### 3.7. Analysis of Tumor Microenvironment and Immune-Related Cells

CIBERSORT was performed to explore the infiltration abundance of 22 immune cells. It was found that resting mast cells, naïve B cells, resting dendritic cells, and monocytes were highly infiltrated in the high-risk groups while resting NK cells, macrophages M0, and T cell follicular helpers significantly infiltrated the low-risk groups (*p* < 0.05, Figure 5D). We compared the immune-cell enrichment scores of two subgroups with ssGSEA. The type I IFN response, type II IFN response, and type I IFN response were enriched in the high-risk group, while for the activity of checkpoint molecules, the scores of cytolytic activity, inflammation promotion, T cell co-inhibition, and T cell co-stimulation, the opposite was true. In terms of the antigen presentation process, activated dendritic cells, APC co-inhibition, and MHC class I varied significantly between the low-risk and high-risk groups (Figure 5E,F).

### 3.8. Crosstalk between Cancer and Immune Cells Based on Necroptosis

**Figure 6:**
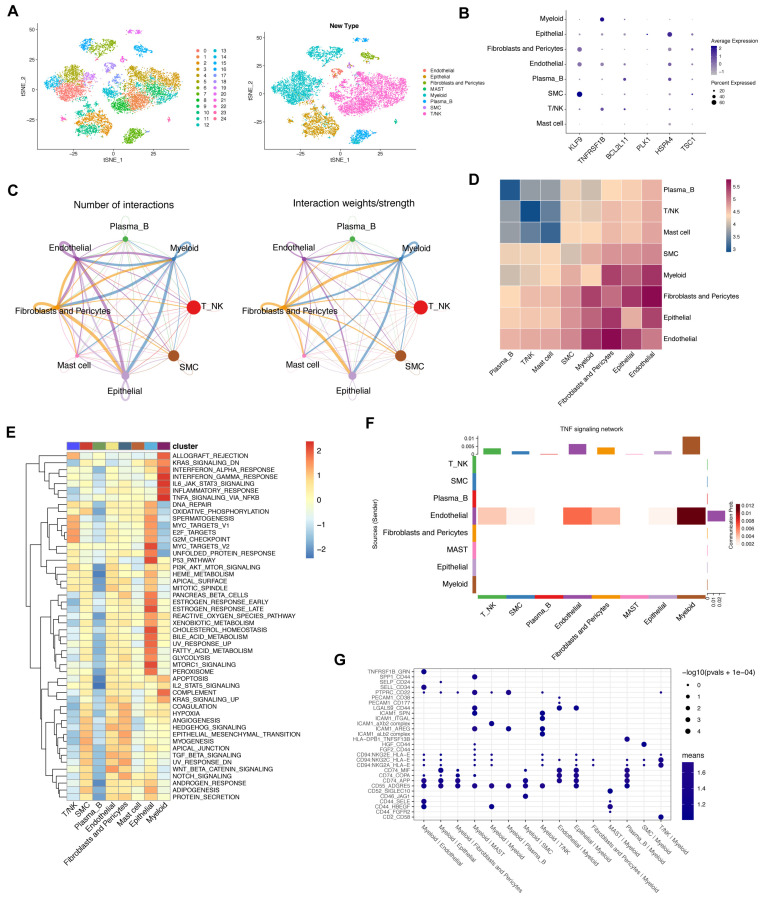
Cell–cell interaction analysis based on single-cell data. (**A**). A total of 12567 cells containing 3 primary gastric cancer samples in GSE163558 were clustered into different types of gastric cancer cells by “Seurat”. (**B**). The 6 prognostic necroptosis-related genes were expressed in different types of cells in gastric cancer. (**C**,**D**). Cell–cell interaction analysis was carried out via “CellChat” and “cellphonedb”. (**E**). GSEA analysis between different types of cells in gastric cancer. (**F**). Ligand–receptor analysis of TNF pathway in different types of cells in gastric cancer. (**G**). Connection probability of main signaling pathways in different types of gastric cancer cells.

To identify the role of NRGs in the TME of GC, single-cell analysis was conducted in the GSE163558 dataset comprised of three primary gastric cancer samples of 12567 cells. Non-linear dimensionality reduction (t-SNE) was applied to cluster the cells into eight subtypes according to the specific cell markers (Figure 6A). The expression levels of six NRGs in TME were investigated. As shown in Figure 6B, KLF was mainly expressed in SMC, TNFRSF1B was mainly expressed in myeloid cells, BCL2L11was mainly expressed in plasma B cells, PLK1 and HSPA4 were mainly expressed in epithelial cells, and TSC1was mainly expressed in fibroblasts and pericytes cells. Cell–cell communications and the cell–cell interaction network between cancer and immune cells were explored by CellChat and cellphonedb and are depicted in Figure 6C,D. Pathway analysis among different cell types showed that the TNF pathway (including TNFRSF1B) could trigger necroptosis in myeloid cells (Figure 6E). Our correlation analysis verified the above results (Figure 6F,G). In summary, our results revealed that necroptosis-related genes could shape the unique TME of GC.

### 3.9. Analysis of Glycolysis, Risk Score, and Immune Cells

**Figure 7:**
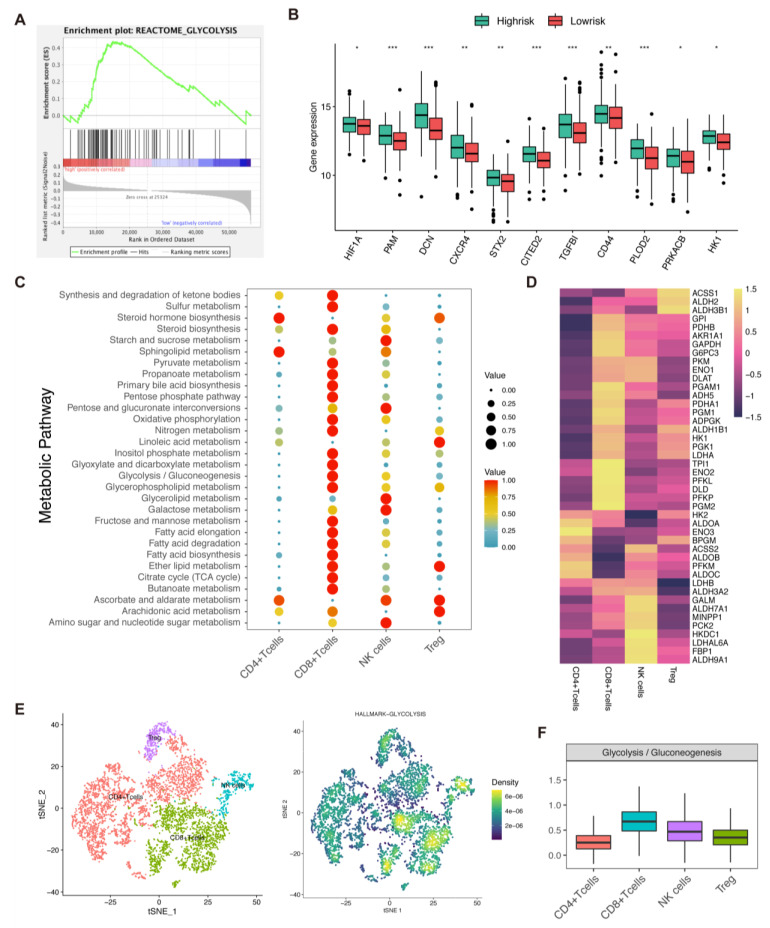
Metabolic status activity of gastric cancer patients. (**A**). GSEA analysis of metabolic-related gene sets. (**B**). Differential analysis of glycolysis-related genes between two risk groups (ns = non-significant; *, ** and *** = significant at 0.05, 0.01 and 0.001 levels, respectively). (**C**,**D**). The average metabolic gene expression and median metabolic pathway score of T and NK cells. (**E**,**F**). Single-cell glycolysis activity of T and NK cells.

We found that the metabolic status of cancer and immune cells was fundamentally different. GSEA analysis showed that REACTOME-GLYCOLYSIS was enriched in the high-risk group (Figure 7A). In addition, glycolysis-related genes were upregulated in the high-risk group (Figure 7B). Analysis was then conducted to investigate the metabolic status of immune cells. We extracted T and NK cell subtypes for further analysis and subsequently examined the association between the metabolic pathways and T/NK cells. CD8 T cells showed preferential activation in classic metabolic pathways including glycolysis (Figure 7C). Further clustering of all glycolysis-associated genes also provided a strong metabolic preference associated with CD8 T cells (Figure 7D). At the same time, we discovered that the CD8 T cell exhibited a glycolysis signature with greater significance than other immune cells (Figure 7E,F).

### 3.10. In Silico Drug Sensitivity Analysis and In Vitro Drug-Sensitivity Investigations

We began by screening the potential drug sensitivity and risk genes from the CTRP and GDSC databases. The predictive NRGs correlated with the sensitivity of several chemotherapeutic and target therapy compounds commonly used to treat advanced tumors (Figure S3A). Further exploration of 161 prodrugs with therapeutic potentialities was conducted between the two risk groups. It was found that the IC50 was significantly different in drugs associated with pathways altered in our previous functional analysis, such as TGF-beta, focal adhesion, hedgehog signaling, and ATP-dependent transmembrane transport complex pathways. Patients in the low-risk group were more sensitive to most drugs, including Rapamycin, Gemcitabine, Phenformin, and OSI-027, which could potentially disrupt glycolysis pathways (Figure S3B and Table S5).

**Figure 8:**
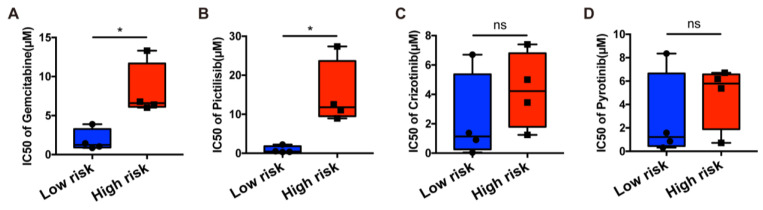
In vitro drug-sensitivity investigations. (**A**–**D**). The high-risk group tended to show lower sensitivity to drug therapy compared with the low-risk group (ns = non-significant; * = significant at 0.05 levels).

Next, we validated drug sensitivity via an in vitro experiment. Eight gastric cancer cell lines (SNU601, SNU668, KATO-III, MKN45, NUGC-4, N87, HGC27, and AGS) were assigned to the low- or high-risk group according to the mRNA expression levels of six necroptosis-related genes based on RNA-seq data on TRON Cell Line Portal (http://celllines.tron-mainz.de, accessed on 18 September 2022) (Table S6). Four cell lines, SNU601, SNU668, KATO-III, and MKN45, showed lower risk than the other four cell lines according to our prediction signature. The cytotoxicity of four drugs in eight gastric cancer cell lines was assessed via the CCK8 assay. Cell lines tended to be more hypersensitive in the low-risk group, especially in gemcitabine and Pictilisib (*p* < 0.05). No difference in the IC50 was observed between the two risk groups in terms of crizotinib and pyrotinib sensitivity (Figure 8A–D). All the above in vitro results confirmed our previous database predictive analysis.

### 3.11. Necroptosis-Related Gene and Cell Line Proliferation and Migration In Vitro

**Figure 9:**
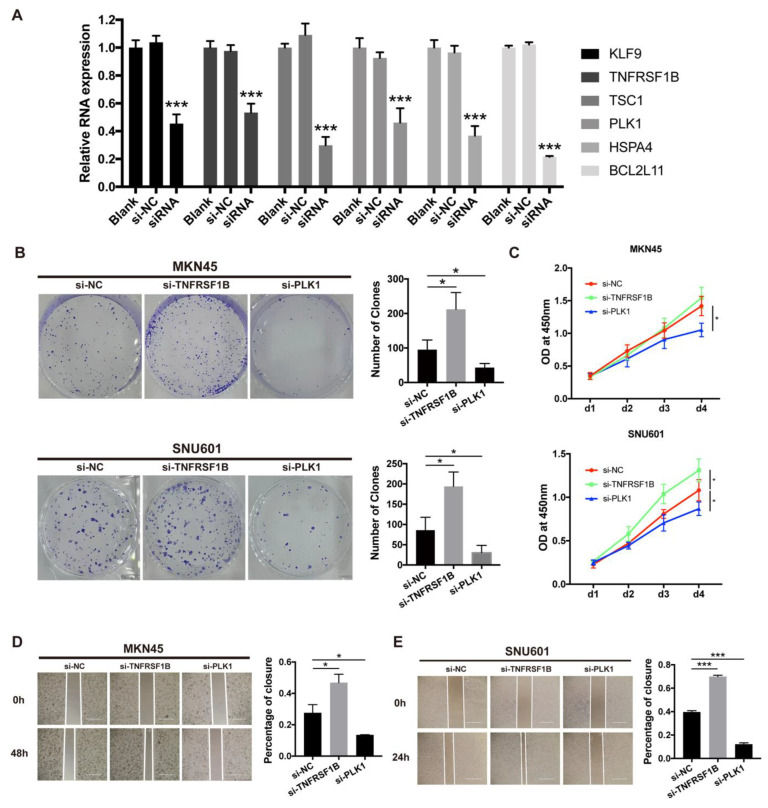
In vitro experiments. (**A**). qRT-PCR to evaluate the level of mRNA expression levels of 6 necroptosis-related genes 2 days after transfection. All siRNA sequences could result in significant decrease in mRNA expression. (**B**). Colony formation assay. Cells with reduced TNFRSF1B and PLK1 expression exhibited a significant change in the numbers of colonies when compared with the siRNA negative control (NC) group. (**C**). CCK8 assay. After TNFRSF1B and PLK1 expression knockdown, the cells altered function in viability. (**D**). Scratch wound healing assay. A significant change in wound healing rate was observed in cells with decreased expression of TNFRSF1B and PLK1. (**E**). In vitro drug-sensitivity investigations. The high-risk group tended to show lower sensitivity to drug therapy compared with the low-risk group (ns = non-significant; * and *** = significant at 0.05 and 0.001 levels, respectively).

The mRNA transcript levels of each necroptosis-related gene in MKN45 cancer cells treated with the corresponding siRNA for 48h were found to decrease significantly when compared with those untreated cells by qRT-PCR (Figure 9A). Cells with reduced TNFRSF1B and PLK1 expression exhibited a significant change in the numbers of colonies in the colony formation assay compared with the siRNA negative control (NC) group (Figure 9B). Although we found that TNFRSF1B knockdown had only a slight impact on proliferation abilities, down-expression of TNFRSF1B dramatically promoted the migration capacity of cancer cells MKN45 and SNU601. Meanwhile, the CCK8 assay showed that the proliferation abilities were obviously decreased by the PLK1 knockdown. A significantly slower scratch wound healing rate was observed in cells with decreased expression of the PLK1 gene (Figure 9C,D). The other four genes showed no significant effect on cell function after transfection. These findings were confirmed by examining the respective gastric cell line, such as AGS and HGC27 (Figure S4).

## 4. Discussion

The mortality rate of GC ranked in third place worldwide [1]. With more than half of patients diagnosed with advanced GC, it requires feasible and effective therapy in a personalized multimodal setting. Studies supported the vital role of necroptosis in oncogenesis, immune infiltration, and antitumor response in GC [23,24]. However, the molecular mechanism of necroptosis regulators in the prognosis and potential of GC has yet to be fully explored. Currently, the emergence of immunotherapy has revolutionized the management of advanced solid tumors including GC. Nevertheless, increasing numbers of GC patients whose cancer progressed after currently available treatments emphasized the importance of molecular treatment options.

In the present study, we began by evaluating the importance of necroptosis in GC. The differentially distributed necroptosis-related genes between tumor tissues and normal tissues were used to construct a prognostic six necroptosis-related genes signature. GC patients were then assigned to the low- or high-risk group. The risk score was validated as an independent prognostic factor of the OS. Patients with a high-risk score had worse prognostic outcomes and a higher grade of pathological characteristics relative to the low-risk subgroup. Next, we identified a necroptosis-related nomogram prediction model. The nomogram prediction model provided satisfactory observations of the 1–3-year survival rate and demonstrated stability and accuracy.

Next, we explored the mechanisms leading to better clinical outcomes in the low-risk subgroup. According to the ESTIMATE algorithm, patients in the low-risk score subgroup had a higher immune score—in other words, they possessed a higher abundance of immune cells. In addition, the CIBERSORT analysis yielded the same results. A previous study showcased a positive relationship between tumor immune infiltrate and patients’ prognosis [25,26]. Our results illustrated that CD8+ T cells, TH1 cells, and TILS were upregulated in the low-risk group. The infiltration of activated CD8+ T cells, which were the most important antitumor effector cells, was associated with favorable survival outcomes in malignancy [27,28]. TH1 cells could promote CD8+ T cells priming and expansion via the TH1-related cytokine, and thus enhance the antitumor effectiveness of CD8+ T cells [29]. Evidently, immune responses generated against neoantigens based on the peptide epitopes bind to specific MHC molecules [30]. Additionally, coupled with the poor enrichment of MHC-I, T cell co-stimulation, and inflammation promotion, the immune activity to GC cells was suppressed in the high-risk group in our study.

The low-risk group also possessed the independent characteristics of a gene signature which reflected defects in POLE and unique CNVS associated with the DNA damage repair pathway. Defects in POLE contributed to genomic stability during normal DNA replication [22]. Genomic instability of the tumor potentially contributed to inflammatory TME [31]. It was reported that the defective POLE gene generated sensitivities in tumors’ response to checkpoint immunotherapy [32]. Furthermore, low-risk patients also presented with higher TMB. The response rate was higher in the high-TMB group treated with checkpoint inhibitors in patients with advanced solid tumors [31,33]. Thus, patients in the low-risk score subgroup might receive more benefits from immune therapy compared with those in the high-risk subgroup.

The TME component is an important contributory factor to the multigenic process of tumorigenesis. With the application of cell-to-cell communication packages, necroptosis was found to be triggered by the TNF pathway (including TNFRSF1B) in myeloid cells. Similar to the results of our study, activation of the TNF-related pathway was reported to promote the immunosuppressive phenotype and function of Tregs in GC [34]. TNF pathway emerged as the key mechanism of CD8+ T cell-mediated killing and immune evasion from primary NK cells [35]. To sum up, the tumor immune microenvironment of the high-risk group may contribute negatively to the progression of GC.

Metabolic reprogramming, including the remodeling of glucose, plays a pivotal role in the regulation of the biological functions of cancer [36]. Glycolysis pathways were enriched in the high-risk group. The difference in the metabolic status might be one explanation for the differences in prognosis between the two risk groups. Glycolysis is also associated with the alteration of oncogenes [37]. In our study, we found that cancer cells display distinct oncogenic alterations in the two risk groups (e.g., KRAS, PIK3CA, mTOR) that appear to directly induce metabolic changes. Metabolic pathways such as glucose competition regulate immune cells’ differentiation and function in TME [38]. Glycolysis plays a key role in T cell activation. The impeded metabolic status of cytotoxic CD8+ T cells that infiltrated tumors restricted antitumor immunity [39].

The present study demonstrated that TNFRSF1B and PLK1 might modulate cell functions, including proliferation and migration in vitro. TNFRSF1B played a vital role in the regulation of tissue regeneration and was a major TNF-α receptor [40,41]. PLK1 was extensively studied to examine its function in cell cycle regulation [42], and it was involved in the development of various cancers [43]. Therefore, our study validated the importance of necroptosis-related genes. It may also provide greater insight into these genes.

Necroptosis status could also predict drug sensitivity in GC. Thus far, only three molecular biomarkers are demonstrably effective in targeted therapies in GC patients [44]. The CTRP and GDSC database was applied to identify chemotherapy and small-molecule drugs for GC patients. Drugs associated with glycolysis disruption were also investigated. According to changes in the values of estimate IC50, the high-risk group tended to show lower sensitivity to drug therapy compared to the low-risk group. This may be due to the alterations in pathway analysis and metabolic status of the two risk subtypes. It is increasingly evident that a higher glycolytic rate could contribute to drug resistance [45]. The inhibition of glycolysis could convert tolerogenic cancer cells into immunogenic phenotypes [46]. For instance, the PI3K/Akt/mTOR pathway is significantly activated in aerobic glycolysis and is correlated with immunosuppressive TAMs [47,48]. Our in vitro results verified the above mechanism, which indicated that PI3K inhibitors and gemcitabine were more sensitive in the low-risk group. Thus, based on the gene status and our risk score predictive analysis, doctors might choose a potential treatment scheme for GC patients, and these researched drugs might also possess efficacy in clinical practice.

## 5. Conclusions

We identified two risk necroptosis statuses of GC and established a robust necroptosis prognostic model. Different necroptosis statuses induced distinct heterogeneity in GC patients at multiple levels, including pathological status, alternation in genomics, tumor microenvironment, metabolic status, sensitivity to drugs, and overall clinical survival. GC patients with a low-risk score might obtain more benefits from immune therapy. Meanwhile, our model could predict each patient’s susceptibility to specific chemotherapy or small-molecule drugs, as well as the patient’s overall survival. The above findings might facilitate a better understanding of the treatment potential and prognosis of GC patients.

## Data Availability

The original contributions presented in the study are included in the article. Further inquiries can be directed to the corresponding authors.

## Supplementary Materials

The following supporting information can be downloaded at: https://www.mdpi.com/article/10.3390/biom13010101/s1, Figure S1: The selection of candidate genes; Figure S2: Clinical characteristics comparison and Functional Enrichment Analysis between two risk groups; Figure S3: In silico drug sensitivity analysis; Figure S4: In vitro validation experiments of AGS and HGC27 cell lines; Table S1: Comparison of clinical-pathological characteristics between TCGA-STAD cohort and GSE84437 cohort; Table S2: Sequences of siRNAs; Table S3: Sequences of primers; Table S4: Information of drugs; Table S5: Comparison of the predicted IC50 of 15 selected drugs between low and high risk group by in silico drug sensitivity analysis; Table S6: Risk stratification of 8 gastric cell lines.
