# Supplementary material for: Necroptosis Related Genes Predict Prognosis and Therapeutic Potential in Gastric Cancer
Source: Biomolecules. 2023 Jan 4;13(1):101. doi: 10.3390/biom13010101 (PMC9856014; doi:10.3390/biom13010101)
Supplement: Supplementary file 1 [file biomolecules-13-00101-s001.zip › biomolecules-2070139-supplementary.pdf]

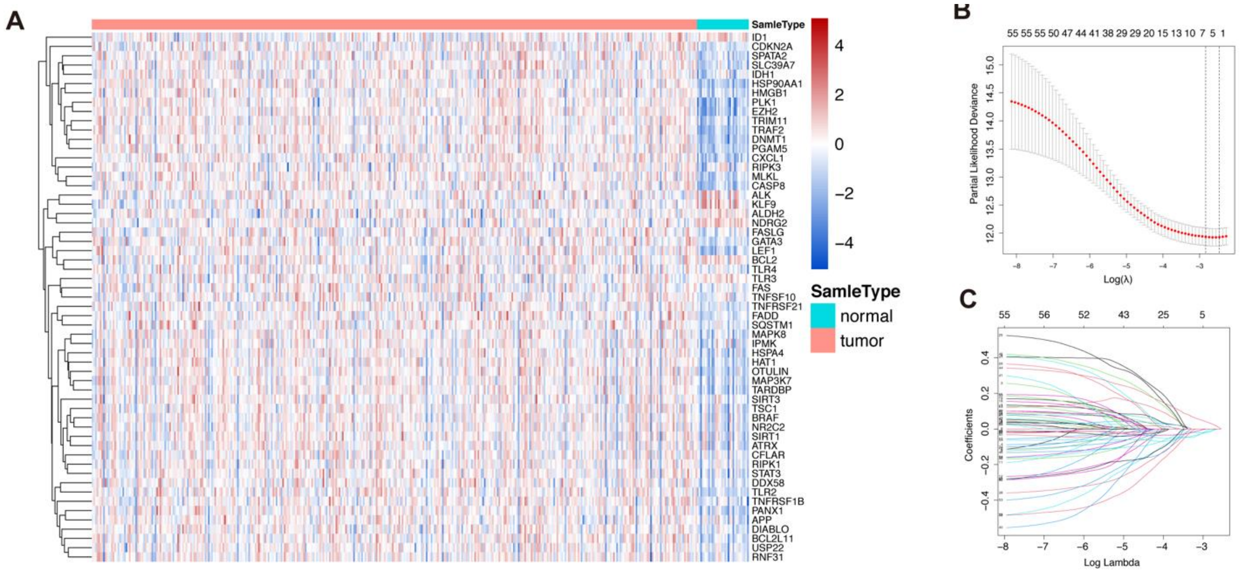

Figure S1. The selection of candidate genes. (A). Heatmap of differential expressed necroptosis-related genes. (B-C). Candidates for constructing the prognostic model with LASSO-Cox regression analysis.

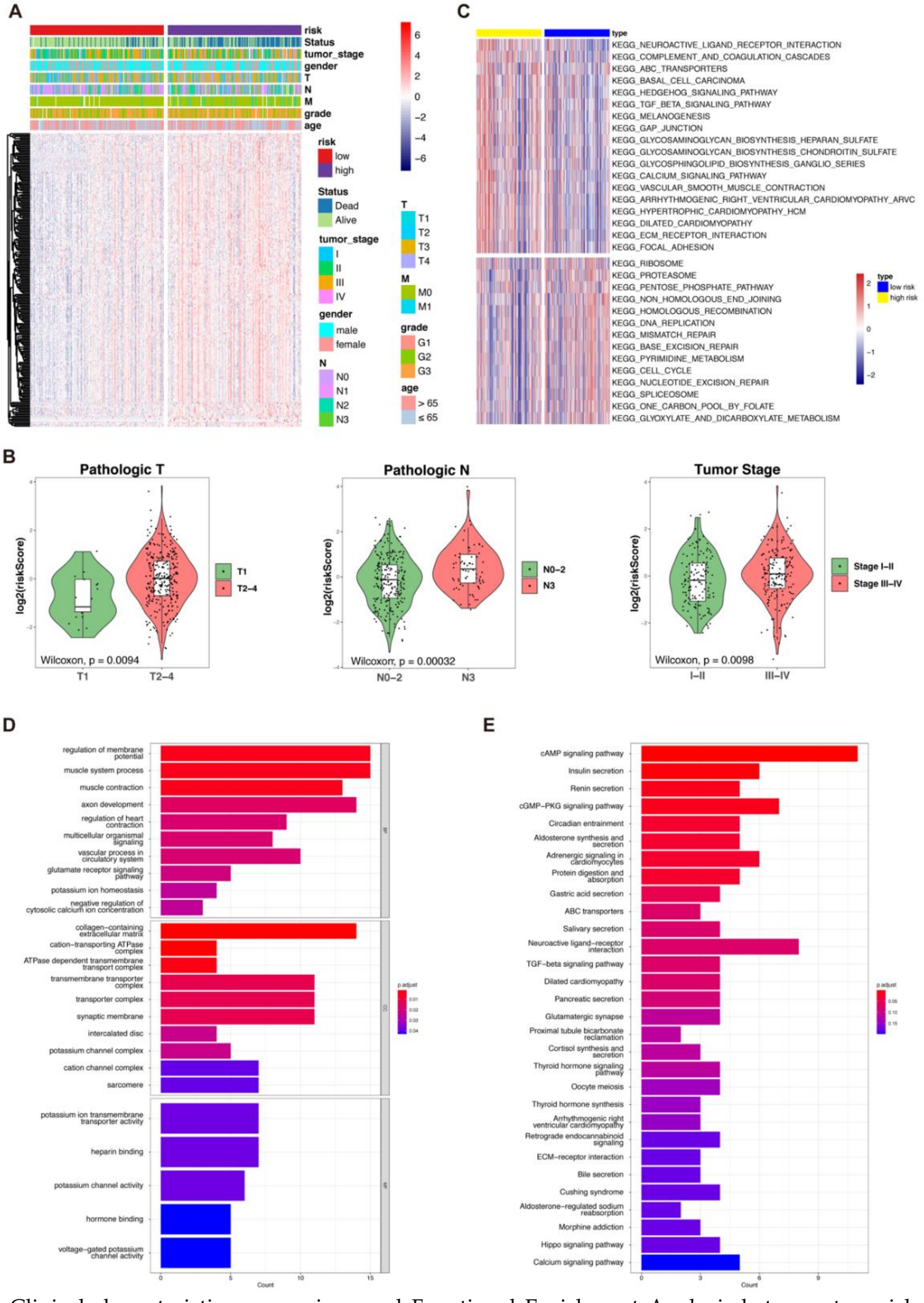

Figure S2. Clinical characteristics comparison and Functional Enrichment Analysis between two risk groups. (A). Differential expressed genes between high and low risk groups in TCGA-STAD cohort. (B). Comparison of risk scores stratified by pathologic T, pathologic N and tumor stage. (C). GSVA analysis of KEGG pathways. (D-E). Functional analysis of DEGs between two risk groups.

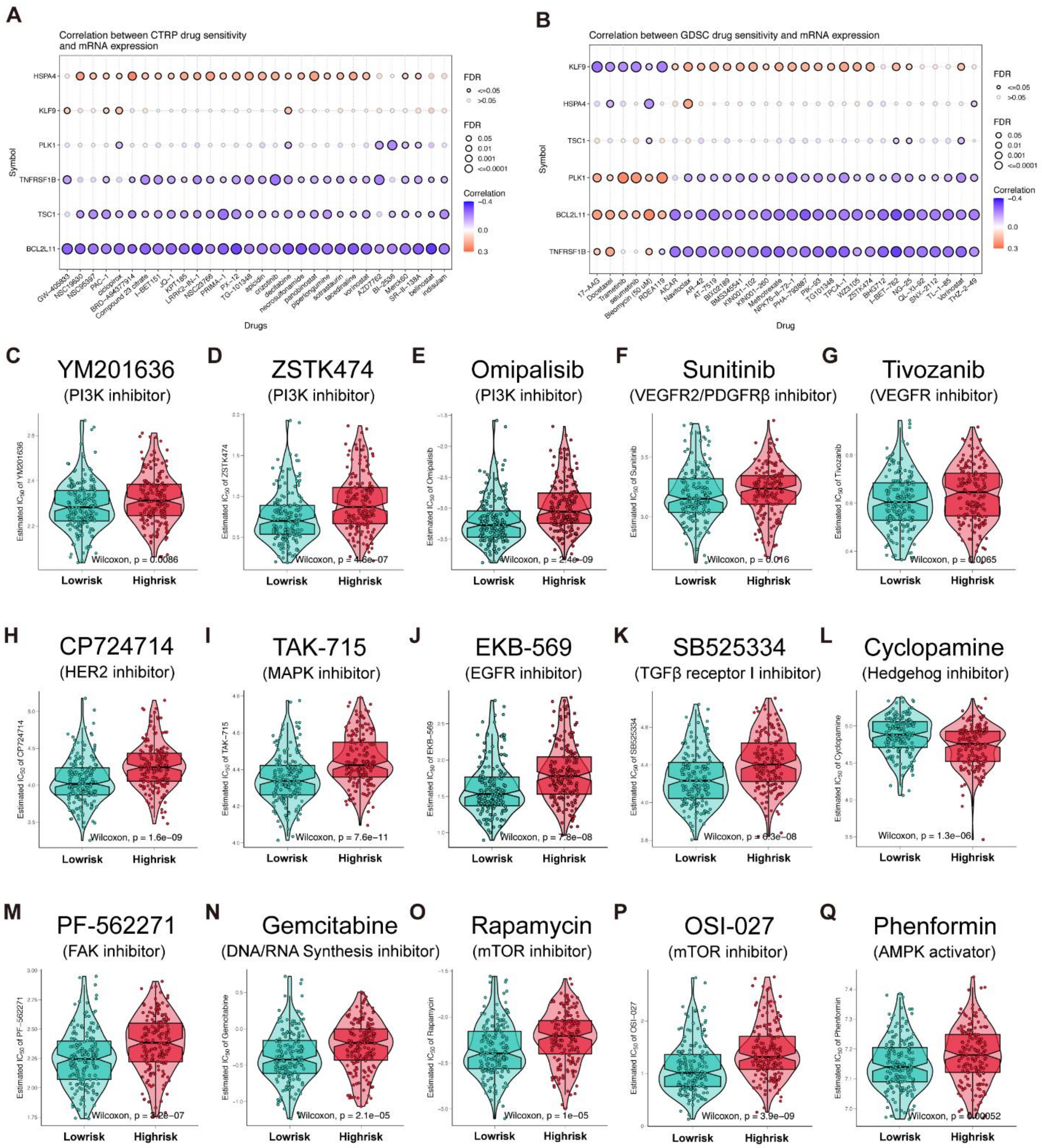

Figure S3. In silico drug sensitivity analysis. (A-B). Correlation between drug sensitivity of GDSC/CTRP and necroptosis-related genes expression. (C-Q). Comparison of clinical chemotherapeutic response between the subgroups predicted by estimated IC50.

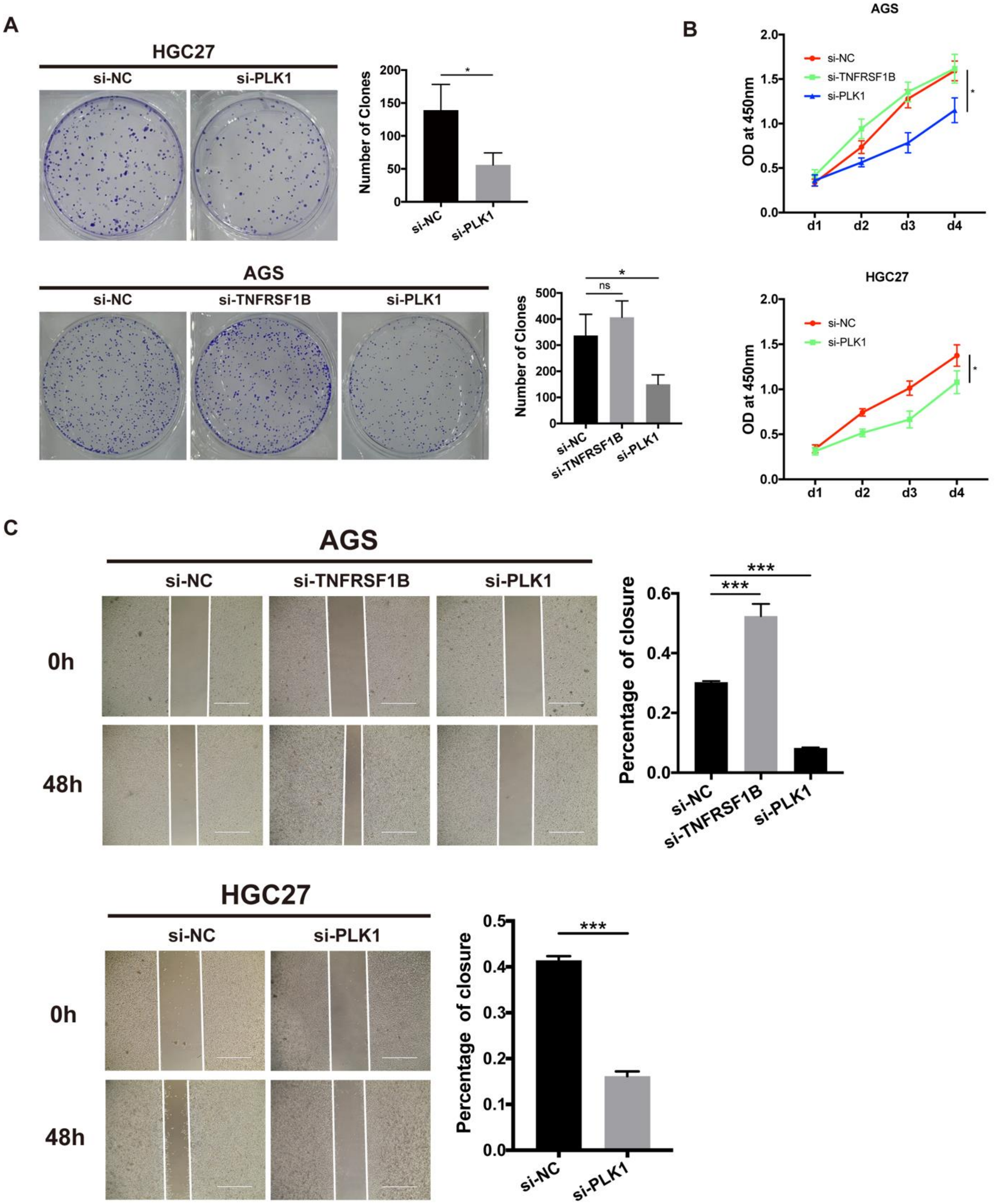

Figure S4. *In vitro* validation experiments of AGS and HGC27 cell lines. (A). Colony formation assay; (B). CCK8 assay; (C). Scratch-wound healing assay.

Table S1. Comparison of clinical-pathological characteristics between TCGA-STAD cohort and GSE84437 cohort

	TCGA-STAD cohort n=350	GSE84437 cohort n=433	*P*
Age, median [min, max]	67.0 [35.0, 90.0]	62.0 [27.0, 86.0]	
≤60	117(33.4%)	194(44.8%)	0.002
>60	230(65.7%)	239(55.2%)	
Unknown	3(0.8%)	0(0)	
Gender (n, %)			
Male	226(64.6%)	296(68.4%)	0.263
Female	124(35.4%)	137(31.6%)	
OS Status (n, %)			
Alive	204(58.3%)	224(51.7%)	0.061
Dead	145(41.4%)	209(48.2%)	
Unknown	1(0.3%)	0(0)	
OS time (months), median [min, max]	15.8 [0.03, 124]	70.0 [1.00, 161]	-
T (n, %)			<0.001
T1	16(4.6%)	11(2.5%)	
T2	74(21.1%)	38(8.8%)	
T3	161(46.0%)	92(21.2%)	
T4	95(27.1%)	292(67.4%)	
Unknown	4(1.1%)	0(0)	
N (n, %)			<0.001
N0	103(29.4%)	80(18.5%)	
N1	93(26.6%)	188(43.4%)	
N2	72(20.6%)	132(30.5%)	
N3	71(20.3%)	33(7.6%)	
Unknown	11(3.1%)	0(0)	
M (n, %)			-
M0	312(89.1%)	NA	
M1	23(6.6%)	NA	
Unknown	15(4.3%)	NA	
TNM stage (n, %)			-
I	46(13.1%)	NA	
II	110(31.4%)	NA	
III	145(41.4%)	NA	
IV	35(10.0%)	NA	
Unknown	14(4.0%)	NA	
Tumor Grade (n, %)			-
G1	9(2.6%)	NA	
G2	125(35.7%)	NA	
G3	207(59.1%)	NA	
Unknown	9(2.6%)	NA	

Table S2. Sequences of siRNA

name	Sequneces
si-PLK1#1	sense: 5'-CCCUCACAGUCCUCAAUAATT-3' antisense: 5'-UUAUUGAGGACUGUGAGGGTT-3'
si-PLK1#2	sense: 5'-GGCAACCAAAGUCGAAUAUTT-3' antisense: 5'-AUAUUCGACUUUGGUUGCCTT-3'
si-PLK1#3	sense: 5'-GCCUCAUCCUCUACAAUGATT-3' antisense: 5'-UCAUUGUAGAGGAUGAGGCTT-3'
si-TNFRSF1B#1	sense: 5'-CCGGCUCAGAGAAUACUAUTT-3' antisense: 5'-AUAGUAUUCUCUGAGCCGGTT-3'
si-TNFRSF1B#2	sense: 5'-GUGGUGAACUGUGUCAUCATT-3' antisense: 5'-UGAUGACACAGUUCACCACTT-3'
si-TNFRSF1B#3	sense: 5'-CACAAUGGGAGACACAGAUTT-3' antisense: 5'-AUCUGUGUCUCCCAUUGUGTT-3'
si-HSPA4#1	sense: 5'-GGUCAAGUGACUGUGAAGUTT-3' antisense: 5'-ACUUCACAGUCACUUGACCTT-3'
si-HSPA4#2	sense: 5'-GUGUGAGAAACUCAAGAAATT-3' antisense: 5'-UUUCUUGAGUUUCUCACACTT-3'
si-HSPA4#3	sense: 5'-CCAGGAAUCUGAAGAACGATT-3' antisense: 5'-UCGUUCUUCAGAUUCCUGGTT-3'
si-KLF9#1	sense: 5'-CAGUGUCUGGUUUCCAUUUTT-3' antisense: 5'-AAAUGGAAACCAGACACUGTT-3'
si-KLF9#2	sense: 5'-CCAAGAGCUUGUUGGACCUTT-3' antisense: 5'-AGGUCCAACAAGCUCUUGGTT-3'
si-KLF9#3	sense: 5'-CCCAUCUCAAAGCCCAUUATT-3' antisense: 5'-UAAUGGGCUUUGAGAUGGGTT-3'
si-BCL2L11#1	sense: 5'-CCCAUGAGUUGUGACAAAUTT-3' antisense: 5'-AUUUGUCACAACUCAUGGGTT-3'
si-BCL2L11#2	sense: 5'-CACCCACGAAUGGUUAUCUTT-3' antisense: 5'-AGAUAACCAUUCGUGGGUGTT-3'
si-BCL2L11#3	sense: 5'-GACGAGUUUAACGCUUACUTT-3' antisense: 5'-AGUAAGCGUUAAACUCGUCTT-3'
si-TSC1#1	sense: 5'-GCAAGCCUUUACUCCCAUATT-3' antisense: 5'-UAUGGGAGUAAAGGCUUGCTT-3'
si-TSC1#2	sense: 5'-GCACUCUUUCAUCGCCUUUTT-3' antisense: 5'-AAAGGCGAUGAAAGAGUGCTT-3'
si-TSC1#3	sense: 5'-CCAAAUCUCAGCCCGCUUUTT-3' antisense: 5'-AAAGCGGGCUGAGAUUUGGTT-3'
si-NC	sense: 5'-UUCUCCGAACGUGUCACGUTT-3' antisense: 5'-ACGUGACACGUUCGGAGAATT-3'

Table S3. Sequences of primers

name	Sequneces
PLK1	Forward: 5'-GTGCCTAAGTCTCTGCTGCTCAAG-3' Reverse: 5'-TCAGGCTCAGTCAGGGCTTTCC-3'
TNFRSF1B	Forward: 5'-TGAAACATCAGACGTGGTGTG-3' Reverse: 5'-TGCAAATATCCGTGGATGAAGTC-3'
HSPA4	Forward: 5'-GCAGACACCAGCAGAAAATAAGG-3' Reverse: 5'-TCGATTGGCAGGTCCACAGT-3'
KLF9	Forward: 5'-ACAAGTACCGACCCATCCAGACC-3' Reverse: 5'-GCCGTTCACCTGTATGCACTCTG-3'
BCL2L11	Forward: 5'-GGTCTGCAGTTTGTTGGAGC-3' Reverse: 5'-GCGTTTCTCAGTCCGAGAGT-3'
TSC1	Forward: 5'-ACACACTGGCATGGAGATGG-3' Reverse: 5'-CAACAGGCGTCTTGGTGTTG-3'
GAPDH	Forward: 5'-GTATCGTGGAAGGACTCATGAC -3' Reverse: 5'-ACCACCTTCTTGATGTCATCAT-3'

Table S4. Information of drugs

Drug name	Targeting pathways
Crizotinib	c-Met and ALK
Pyrotinib	EGFR and HER2
Gemcitabine	Glycolysis, DNA synthesis
Pictilisib	PI3K

Table S5. Comparison of the predicted IC50 of 15 selected drugs between low and high risk group by in silico drug sensitivity analysis

Drug name	Targeting pathways	Inhibitory effect *	P value
Gemcitabine	DNA/RNA Synthesis inhibitor	Low risk	>0.001
EKB-569	EGFR inhibitor	Low risk	>0.001
CP724714	ErbB2 (HER2) inhibitor	Low risk	>0.001
PF-562271	FAK inhibitor	Low risk	>0.001
Cyclopamine	Hedgehog inhibitor	High risk	>0.001
TAK-715	MAPK inhibitor	Low risk	>0.001
OSI-027	mTOR inhibitor	Low risk	>0.001
Rapamycin	mTOR inhibitor	Low risk	>0.001
Sunitinib	PDGFR inhibitor	Low risk	0.016
Omipalisib	PI3K inhibitor	Low risk	>0.001
YM201636	PI3K inhibitor	Low risk	0.009
ZSTK474	PI3K inhibitor	Low risk	>0.001
SB525334	TGFβ receptor inhibitor	Low risk	>0.001
Pazopanib	VEGFR inhibitor	Low risk	0.012
Tivozanib	VEGFR inhibitor	Low risk	0.006

*The risk group that show higher sentivity to the selected drug

Table S6. Risk stratification of 8 gastric cell lines

Cell line	Risk score	Risk level
SNU601	36.06318582	Low risk
SNU668	42.25750898	Low risk
KATO-III	17.98906564	Low risk
MKN45	63.38277402	Low risk
NUGC-4	79.40894874	High risk
NCI-N87	80.00936189	High risk
HGC27	102.6027212	High risk
AGS	80.96568954	High risk
